# Prevalence of abnormal cervical cytology among subfertile Saudi women

**DOI:** 10.4103/0256-4947.68550

**Published:** 2010

**Authors:** Dania Al-Jaroudi, Tasnim Z. Hussain

**Affiliations:** From the Reproductive Medicine Unit, Women’s Specialized Hospital, King Fahad Medical City, Riyadh, Saudi Arabia

## Abstract

**BACKGROUND AND OBJECTIVES:**

Since cervical cancer is reportedly the seventh most frequent cancer in women in Saudi Arabia and the eighth most frequent cancer among women aged between 15 and 44 years, we wanted to determine the prevalence of abnormal cervical cytology among subfertile women attending the reproductive medicine unit of a tertiary care center in Saudi Arabia.

**METHODS:**

This was a retrospective, cross-sectional, hospital-based study. A Pap smear was done for 241 of 493 (48.9%) subfertile women from January 2008 through February 2009.

**RESULTS:**

The Pap smear was normal in 166 of 241 patients (67.9%), abnormal in 71 (29.5%), and unsatisfactory for evaluation in 4 (1.7%). According to the revised Bethesda system, epithelial cell abnormality was found in 7 (2.9%), inflammation in 55 (22.8%), and infection in 9 (3.7%) patients. Epithelial cell abnormalities were further classified as atypical squamous cells of undetermined significance (ASC-US) (n=3, 42.8%), atypical squamous cells of high grade (ASC-H) (n=1, 14.3%), low-grade squamous intraepithelial lesion (LSIL) (n=2, 28.5%), and glandular cell abnormalities (AGS) (n=1, 14.3%).

**CONCLUSION:**

The high prevalence of abnormal cervical cytology in our subfertile women accentuates the need for screening in patients eligible for in vitro fertilization. In addition, a well-organized screening program for cervical cell abnormalities at the national level should be implemented to allow identification of subfertile women at risk so that potentially life-saving measures can be undertaken early.

Cervical cancer, known for its mortality and morbidity, is the second most common cancer in women worldwide, with an estimated 493 000 new cases and 274 000 deaths in 2002.^1^ Human papilloma virus (HPV), especially HPV 16 and 18, plays a major role in cervical cancer etiology.^2^ Risk factors include age, parity, marital status, age at first intercourse, number of sexual partners,^3^–^5^ and cigarette smoking.^5^–^8^ Three-fourths of women will be infected with HPV at least once in their lifetime, and abnormal cervical cytology is a common presentation.^9^ Early detection of cervical cell abnormalities by Papanicolaou (Pap) smear has reduced the risk of cervical cancer development by allowing timely response to abnormal changes in cervical cytology. Detection rates of squamous cell abnormalities improved with the use of liquid-based thin prep testing of cervical cells, which was approved by the US FDA in 1996 and has now become standard practice internationally.^10^

Cervical cancer is the seventh most frequent cancer in women in Saudi Arabia and the eighth most frequent cancer among women aged between 15 and 44 years.^11^ The crude incidence rate of cervical cancer in Saudi Arabia was estimated at 2.7/100 000 in 2002, compared with a world rate of 16.0/100 000.^11^ Current estimates indicate that every year 271 Saudi women are diagnosed with cervical cancer, with 68 (25.1%) cases occurring in women of child-bearing age. Of these 271 women, 143 (52.8%) will die due to the disease, including 27 (39.7%) who are of child-bearing age.^11^ Although the population-based mortality in Saudi Arabia of 1.4/100 000 is much less than the 8.9/100 000 reported for the rest of the world, the fact that over one-third of Saudi women of reproductive age who have cervical cancer will die from the disease contrasts starkly with the 8.9% of their counterparts worldwide.^11^

In the few published studies from Saudi Arabia the prevalence of epithelial cell abnormalities in the western region of the country has been variously reported to be 5.0%,^12^ 1.5%,^13^ and 2.2%.^14^ In addition, there are few publications on the prevalence of abnormal cervical cytology in subfertile women attending fertility clinics.^15^ This paucity of data is related to the lack of a national screening program for cervical cancer in Saudi Arabia. We investigated cervical abnormalities in subfertile women attending our clinic to provide information that may help promote interest in establishing a national program.

## METHODS

A retrospective, cross-sectional, chart review of Pap smear data was performed for all women presenting with subfertility at the Reproductive Medicine Unit (RMU), Women’s Specialized Hospital, King Fahad Medical City, Riyadh, Saudi Arabia, during the 14-month interval from January 2008 through February 2009. A total of 493 women attended the clinic during that interval. Routine cervical smear is offered to all patients as part of their subfertility workup. Liquid-based preparation is used to acquire the samples, which are analyzed in the pathology department according to the revised Bethesda system (2001). The samples are sent to the hospital laboratory as a routine procedure and are read by different pathologists according to the lab protocol. Data acquired from the medical records included Pap smear results, patient age, duration and description of subfertility, medical and surgical history, and complaints (if any) at the time of the initial presentation.

## RESULTS

A cervical smear was taken from 241 of the 493 patients (48.9%); the rest of the patients were not willing to undergo screening. The 241 patients who had cervical smears taken had a mean age of 30.1 years (standard deviation, 5.6 years; range 18-43 years). One hundred forty-nine (61.8%) had primary subfertility, while 92 (38.2%) had secondary subfertility. Major causes of subfertility were female factor in 97 (40.2%), male factor in 84 (35%), combined male and female causes for 23 (9.5%), and unexplained subfertility in 37 (15.3 %) couples (Table 1). None of the patients had other complaints at the time of initial presentation; all patients denied tobacco use and all were HIV negative.

**Table 1:** Causes of subfertility in study population (n=241).

Subfertility factors	No. of couples	% of total
Anovulation	61	25.3
Tubal factor	17	7.1
Endometriosis	5	2.1
Uterine anomalies	5	2.1
Premature ovarian failure	2	0.8
Others	7	2.9
Male factor	84	34.8
Combined male and female factor	23	9.5
Unexplained	37	15.3

Cervical cytology was normal for 166 (68.9%) patients and abnormal for 71 (29.5%); samples from 4 (1.7%) patients were unsatisfactory for evaluation. Abnormalities included epithelial cell abnormality in 7 (2.9%), inflammation in 55 (22.8%), and infection in 9 (3.7%) patients. Epithelial cell abnormalities included three atypical squamous cells of undetermined significance (ASC-US), one atypical squamous cells of high grade (ASC-H), two low-grade squamous intraepithelial lesions (LSIL), and one glandular cell abnormality (AGS) (Table 2).

**Table 2:** Epithelial cell abnormalities in patients with subfertility.

Age	Subfertility	Duration (years)	Cause	Result	Repeat smear
26	Primary	5	Male	LSIL	Negativea^a^
29	Secondary	4	Anovulation	ASC-US	No repeat smear
26	Primary	2	Anovulation	ASC-US	No repeat smear
31	Secondary	4	Unexplained	AGS	No repeat smear
39	Primary	3	Anovulation	ASC-H	Negativea^a^
30	Primary	4	Anovulation	ASC-US	No repeat smear

a Repeated after 2 months.

All patients with abnormal results were managed according to the type of abnormality present. One patient had bacterial vaginosis and was treated with metronidazole. Other patients had candidiasis and were treated with 100 mg clotrimazole for 6 days. Repeat cultures for patients were negative. All patients with epithelial cell abnormalities were referred for colposcopy and managed according to standard practices. Repeat smears for three patients were negative within 6 months of the initial smear, and the other four women are being managed according to standard practice.

## DISCUSSION

Screening with Pap smear allows earlier detection of cervical cell abnormalities and prompts action according to set guidelines. Screening has been shown to be accompanied by a dramatic reduction in the incidence of invasive cervical cancer.^1^ Unfortunately, fewer than 50% of our patients accepted the offer of routine screening for cervical assessment, which may reflect lack of public awareness of cervical cancer and the related morbidity and mortality. The 29.5% prevalence of abnormal cervical cytology in women eligible for in vitro fertilization (IVF) treatment in our clinic is surprisingly more than that reported by both van Hamont et al^15^ and Lundqvist et al,^16^ who found that abnormal cervical cytology was more prevalent in subfertile women than in healthy controls, with 6.1% of 699 women undergoing IVF showing this abnormality as compared with 3.9% of 77 055 matched controls.^15^–^16^ The Dutch CISOE-A classification of cervical cytology was used in the van Hamont study;^15^ this classification discriminates between normal cytology, borderline nuclear changes, mild dyskaryosis, moderate dyskaryosis, severe dyskaryosis, carcinoma in situ, and carcinoma of the cervix. In another case-control study, 214 women who were having IVF were compared with 197 healthy control women; the prevalence of abnormal cervical cytology in the subfertile women was 2.3%, which is also much less than the 29.5% found in our study. The authors used the CIN (cervical intraepithelial neoplasia) classification system for reporting the Pap smear results;^17^ in contrast to the results of van Hamont et al, the rate of abnormal cervical cytology of 4.1% in the control group in this study was higher than that in women undergoing IVF, but it was still much less than that in our group.^16^

Our study adds to the information from the few reports that address patterns of abnormal cervical cytology among subfertile women. Our evidence would have been stronger and more credible if we had compared the prevalence of abnormal cervical cytology among subfertile women to that in similarly matched fertile controls. Larger studies are needed that compare subfertile women with a control group to elicit the prevalence of abnormal Pap smear in these two populations. Additionally, screening for HPV should be done in women with epithelial cell abnormalities to help identify this potential etiological agent.

In conclusion, the high prevalence of abnormal cervical cytology in our group of subfertile women stresses the need for cervical cytology screening in patients eligible for IVF. This calls for a well-organized screening program at the national level for cervical cell abnormalities, which would allow identification of subfertile women at risk for whom early potentially life-saving measures can be undertaken. Additionally, national patient information programs are required that will create public awareness and promote understanding of the need for cervical cancer screening. At the clinic level, educational programs can include tutorials presented while women are waiting for appointments, distribution of handouts, and information displays.
